# Preclinical safety assessment of modified gamma globin lentiviral vector-mediated autologous hematopoietic stem cell gene therapy for hemoglobinopathies

**DOI:** 10.1371/journal.pone.0306719

**Published:** 2024-07-08

**Authors:** Mohammad Shadid, Archana Shrestha, Punam Malik

**Affiliations:** 1 Aruvant Sciences, New York, NY, United States of America; 2 Division of Experimental Hematology and Cancer Biology, Cincinnati Children’s Hospital Medical Center, Cincinnati, OH, United States of America; 3 Division of Hematology, Cincinnati Children’s Hospital Medical Center, Cincinnati, OH, United States of America; Fudan University, CHINA

## Abstract

Previously, we reported the development of a human ^A^γ-globin gene lentivirus (LV), G^b^G, which expresses high levels of HbF to correct the sickle cell anemia (SCA) phenotype in the Berkeley SCA mouse model, and then modified the γ-globin gene by substituting glycine at codon 16 with aspartic acid in the ^A^γ-globin gene to generate G^b^G^M^ LV. In the present study, we evaluated the long-term safety of human ^A^γ-globin gene carrying G^b^G^M^ LV in wild-type mice after primary and secondary transplants of G^b^G^M^-modified hematopoietic stem cells (HSC) over 18 months. The safety of the G^b^G^M^ bone marrow transplant was assessed by monitoring the effects on body weight, hematology, histopathology, malignancy formation, and survival. Mice transplanted with Mock-transduced and spleen focus forming virus (SFFV) γ-retroviral vector (RV)-transduced HSC served as negative and positive controls, respectively. The mean donor-cell engraftment was comparable across Mock, G^b^G^M^ LV, and SFFV RV groups. There were no significant differences in body weight, clinical signs, immunophenotype, or histopathology in the G^b^G^M^-treated mice compared to controls. Four SFFV RV-treated mice, but none of the G^b^G^M^-treated mice, developed donor-derived, vector-positive lymphomas as demonstrated by flow cytometry analysis and in situ hybridization. These results highlight the safety of the administration of G^b^G^M^ LV-modified HSC with long-term follow-up after primary and secondary transplants in mice. This data supported the initiation of phase 1/2 first-in-human SCA clinical trial in the United States.

## Introduction

Sickle cell disease (SCD) is an autosomal recessive genetic disorder caused by a mutation in the β-globin chain genes leading to sickle hemoglobin production (hemoglobin S or HbS) instead of the normal adult hemoglobin, HbA. The single nucleotide mutation in the 6^th^ codon of the β-globin gene results in the substitution of glutamic acid to valine, thus forming the defective HbS. HbS polymerizes upon deoxygenation, changing the shape of the round RBC to sickle shapes and causing deformation of red blood cells (RBC), microvascular occlusions, ischemia, and multi-organ damage [1].

The Food and Drug Administration approved hydroxyurea for treating adults with SCD. Hydroxyurea induces fetal hemoglobin (HbF) production, which prevents sickle polymer formation and RBC sickling [2]. Although hydroxyurea has a dramatic effect on the SCD phenotype, it alleviates the pain and reduces the number of vaso-occlusive crisis and blood transfusions for SCD patients; it is not a curative approach as it requires chronic daily dosing, frequent monitoring, and results in significant compliance issues. Alternatively, allogeneic bone marrow transplantation (BMT) from HLA-matched donors has been effectively used as a curative option for SCD [3–5]. However, not all patients are eligible for BMT due to difficulty in finding human leukocyte antigens (HLA)-matched donors, which are available only to about 15% of patients. Haploidentical transplants are currently being tried but carry a high risk of graft-versus-host disease (GVHD) which can be life-threatening, or graft rejection, resulting in the re-acquisition of SCD [6]. Recently, ex vivo lentiviral vector (LV)-mediated transduction of autologous CD34+hematopoietic stem cells (HSC) as gene therapy has achieved significant clinical benefit for many inherited diseases, such as X-linked severe-combined immunodeficiency, adenosine deaminase deficiency, adrenoleukodystrophy, Wiskott-Aldrich syndrome, β-thalassemia, and SCD [7–13]. However, one of the risks of lentiviral gene therapy has been the possibility, albeit rare, of developing a hematological malignancy due to insertional oncogenesis (IO) [14]. It was reported that 4 out of 20 patients with severe combined immune deficiency–X1 treated with a γ-retroviral vector (RV) carrying the IL-2R gene transfer into autologous CD34+ bone marrow cells developed leukemia [15]. The leukemia was attributed to the higher propensity of RV to integrate near gene promoters, and the viral enhancers in the long terminal repeats (LTR) of RV enhance gene expression of the transgene and surrounding genes ubiquitously; this resulted in overexpression of the proto-oncogene LIM domain-only 2 (LMO2), where the RV had integrated. It was reported that the risk of hematological malignancies can be mitigated by using LV, engineered with a self-inactivating design that results in the deletion of enhancers in the LTR in the integrated provirus and the use of internal promoters to drive expression of the therapeutic transgene [16]. Furthermore, LV-carrying globin genes have an additional safety feature of erythroid-specific internal enhancers and promoters, which are minimally active in hematopoietic stem and progenitor cells (HSPC), and then it is cleared from the cells when the erythroid cells lose their nucleus upon differentiation [17].

We have previously reported the development of a human ^A^γ-globin gene LV, G^b^G, which expresses high levels of HbF to correct the sickle cell anemia (SCA) phenotype in the Berkeley SCA mouse model following reduced intensity conditioning [18]. The transduction of the γ-globin gene resulted in increased expression of HbF within RBCs and correlated with a reduction in reticulocyte counts and an increase in hemoglobin concentration. Subsequently, this significantly improved survival in the G^b^G-treated mice [18]. Furthermore, the safety of the G^b^G vector was evaluated in juvenile pigtailed macaque yielded stable HbF expression in RBCs for three years with no evidence of IO [19]. We have subsequently modified the γ-globin gene by substituting glycine at codon 16 with aspartic acid (G16D) in the ^A^γ-globin gene to generate G^b^G^M^ LV. This change is expected to improve the activity of the ^A^γ-globin, but the vector backbone remains intact.

In the present study, we describe the long-term safety of G^b^G^M^ in wild-type mice after primary and secondary transplants. The safety of G^b^G^M^ was assessed by monitoring the effects on body weight, hematological parameters, histopathology of selected organs, malignancy formation, and survival. Furthermore, the development of vector-positive hematological malignancies in the treated animals was evaluated after primary and secondary transplants using flow cytometry and in situ hybridization to determine the origin of the malignancies. Two different mouse strains were used to take advantage of the different HSPC signatures between the two mice strains (CD45.1 and CD45.2). Therefore, we were able to track the source of malignancies and determine that they were not caused by the G^b^G^M^ treatment. This study was used to support the IND filing of the G^b^G^M^ LV for the treatment of SCD and initiate phase 1/2 first-in-human SCA clinical trial in the United States.

## Materials and methods

### Test and control articles

The test article is a mutant Aγ-globin gene carrying LV G^b^G^M^ that expresses human γ-globin with a point mutation at exon 1. This point mutation was made as in our previously published G^b^G LV [18]. The RV encodes green fluorescent protein (GFP), and the plasmid pRSF91GFP.pre* was kindly provided by Dr. Christopher Baum and used by the CCHMC’s Vector Production Facility to produce the pRSF9 l GFP.pre* vector pseudotyped with the ecotropic envelope (pRSF91GFP.pre*/Eco). This vector is a spleen focus forming virus (SFFV) long terminal repeat driven RV that was used as a positive control in the insertional mutagenesis study described by Modlich et al. [20].

### Animals

C57Bl/6 and BoyJ 6–8-week-old mice were purchased from Jackson Laboratories. The mice were 8–12 weeks old at the time of transplantation.

### Lineage-depletion and cellular transduction

Bone marrow cells were harvested from C57BL/6 (CD45.2) wild-type mice, processed, and purified by density gradient centrifugation. A lineage-depletion was performed to enrich for hematopoietic stem and progenitor cells. The enriched Lineage negative (Lin-) cells from donor mice were then stained using Sca-1 PE (BD Biosciences, San Jose, CA; cat.no. 553108), c-kit APC (BD Biosciences, San Jose, CA; cat.no. 553356) and Streptavidin FITC (BD Biosciences, San Jose, CA; cat.no. 554060). The Lin- Sca 1^+^ c-kit^+^ (LSK) cells were sorted and then equally split between the three experimental groups. LSK cells were cultured in StemSpan medium (Stem Cell Technologies, Vancouver, BC, Canada, cat. no. 09650) with 1% penicillin/streptomycin (Cell gro 30-002-66) containing 2% FBS (Atlanta, S12450), mSCF (50 ng/ml; Peprotech Rocky Hill, NJ; cat. no. 213–13), mlL3 (10 ng/ml; Peprotech Rocky Hill, NJ; cat. no. AF-250-03), LDL (Sigma, St Louis, MO) and dNTP (Qiagen Valencia, CA; cat. no. 1005631) throughout pre-stimulation and transduction. The three test groups for this study were LSK bone marrow cells obtained from C57BL/6 mice that were: I) transduced ex-vivo with the G^b^G^M^ lentiviral vector (G^b^G^M^ -test group); 2) cultured in the same medium without lentiviral vector (Mock/negative control group); and 3) transduced with pRSF91GFP.pre*/Eco (SFFV RV/positive control group). Cells were genetically manipulated using a lentiviral transduction research protocol (Mock and G^b^G^M^). Briefly, LSK cells were pre-stimulated for approximately 16 hours and subsequently co-cultured with test vector (G^b^G^M^) or media (Mock) overnight. After 7–8 hours G^b^G^M^ transduced cells underwent a second round of lentiviral transduction using fresh G^b^G^M^ vector. These lentiviral transduced cells were harvested and formulated for transplant the next day. Bone marrow LSK cells for y-RV transduction (SFFV RV y-RV) were pre-stimulated for 42 hours. Cells were then harvested and transduced twice over two consecutive days using pRSF91GFP.pre*/Eco (SFFV RV γ-RV). For each γ-RV transduction, cells were exposed overnight to the vector in retronectin-coated tissue culture plates that had been pre-loaded twice with vector. The day after the second transduction (96 hours later), cells were harvested, formulated for transplant, and sampled for certification.

### Preparation of dosing solutions and dosing

#### Primary recipients

Transduced LSK cells were harvested, counted, and centrifuged. The supernatant was removed, and cells were re-suspended in 3000 μI PBS, which equals 300 μI cell solution per recipient mouse (+ 10% additional volume for testing), filtered and placed on ice. The donor to recipient animal ratio was 1:1, and approximately 10% of the cells were removed for transduced cell characterization. Sterility testing was initiated on the day of the transplant. LSK bone marrow cells transduced with test and control vector were tested for vector copy number after 14–16 days of culture to dilute out any non-integrated vector. The target VCN/cell was 0.5–5.0 for cells transduced with the G^b^G^M^ lentiviral vector and SFFV RV/positive control vector. For the SFFV RV/positive control group EGFP expression was also assessed using flow cytometry.

Recipient mice were selected for transplantation based on age, body weight, and gender. Each dose group was age and gender-matched and consisted of five females and five males. On the morning of transplantation, all recipients (B6.SJL-Ptprc^a^ Pepc^b^/BoyJ; termed BoyJ) received a first gamma-irradiation dose of 700 cGy; the second dose of 475 cGy was delivered 3–4 hrs after the first dose. During this time, cells were harvested from donor animals and processed for injection, as described above. Each recipient animal was dosed with 300 μI of formulated cells within 3 hours of the second irradiation dose and tagged with an ear tag containing the animal ID number immediately thereafter.

#### Secondary recipients

At the scheduled time of euthanasia (8 months post-transplant), bone marrow was isolated from primary recipients, spun down, and re-suspended in sterile PBS. Bone marrow from one primary recipient mouse was injected into two secondary BoyJ recipients, and all mice were tagged with an ear tag containing the animal ID immediately, thereafter. Before transplant, the recipient BoyJ mice received a first gamma-irradiation dose of 700 cGy and a second dose of 475 cGy 3–4 hrs after. Each dose group yielded up to 10 male and 10 female secondary recipients.

### Animals husbandry

Male and female BoyJ mice were selected as the test system: 15 male and 15 female primary recipients and 26 male and 26 female secondary recipients were assigned to the study. Animals were removed from the study if the 6- or 7-week peripheral blood analysis indicated that cell transduction or engraftment of gene-modified cells did not meet the acceptance criteria. According to the study protocol, mice with donor cell engraftment ≥30% and VCN ≥ 0.2, as determined at the early 7-week time point, were considered evaluable at the end of the study. Mice were housed in autoclaved polycarbonate cages with waterspouts and micro-isolator tops. Standard bedding and irradiated rodent chow were provided by Vet Services. Males and females were housed separately to avoid injury or death due to fighting and to avoid pregnancy. When mice were transported and housed for irradiation and BM transplantation, cages were provided with autoclaved water in autoclaved sterile bottles.

Donor and recipient animals were observed for a minimum of 6–7 days before dosing, irradiation, or sacrifice. Mice were fed standard mouse feed ad libitum as provided by the CCHMC Veterinary Services except for 7 days before and 2–3 weeks after the recipient mice were gamma-irradiated for bone marrow ablation. During this peri-transplant period, mice were fed regular Doxycycline feed as provided by the CCHMC Veterinary Services. UV-treated and endotoxin-screened municipal water was provided ad libitum. When water bottles were used, water and bottles were autoclaved. Only healthy animals were used for the study. After animals were assigned to study groups, at least one male and one female from each shipment was euthanized. Blood was collected for serological assessment for antibodies against pathogens common to rodents. These animals also received a detailed necropsy to identify any lesions suggesting disease. Serum was analyzed for the presence of Sendai virus (Sendai), pneumonia virus of mice (PYM), Mouse hepatitis virus (MHV), Minute virus of mice (MVM), Theiler’s murine encephalomyelitis virus (TMEV [GDVII]), Reovirus Type 3 (Reo3), Mycoplasma pulmonis (MPUL), Mouse rotavirus (EDIM), Mouse parvovirus (MPV), parvovirus protein (VP-2), and murine norovirus (MNV). CCHMC’s animal research facilities are fully accredited by the Association for the Assessment and Accreditation of Laboratory Animal Care (AAALAC). This study complied with all applicable sections of the Final Rules of the Animal Welfare Act regulations.

### Mouse observations and body weights

Throughout the study, all animals were observed daily by animal care staff for morbidity and mortality. Animals with ruffled fur coats, hunched postures, severe dyspnea, self-mutilation, and/or reluctance to move upon stimulation were considered for humane euthanasia. Additional observations were made by study personnel if clinical signs warranted. Body weights were recorded for all primary and secondary dose groups once weekly until the end of the study.

### Evaluation of engraftment of gene modified cells in primary and secondary mice

At 7- and 17-week post-transplant, peripheral blood was collected from study animals and analyzed by flow cytometry (FACS) and qPCR to evaluate engraftment of gene-modified cells. Blood was collected from the lateral tail vein in an EDTA-containing tube. RBC Lysis was carried out using an ammonium chloride-based method (Pharmlyse, BO), and the cells were divided for qPCR or FACS analysis. Cells for FACS were stained with antibodies against CD45.1 and CD45.2 to distinguish between host and donor cells and against CD 11b, CD3 and B220 for immunophenotypic characterization (Antibodies used: CD45.1-APC, CD45.2-PE, CD 11b-PECy7, CD3-FITC and B220-APC-Cy7; all from BD Biosciences, San Jose, CA; cat.no. 561873, 560695, 552850, 555274, and 552094, respectively).

### Necropsy and tissue harvest

All animals received a complete necropsy, including those sentinel animals indicated for serology testing. Moribund animals identified 4 weeks or later after dosing were humanely euthanized using CO_2_, followed by cervical dislocation. Whole blood was collected in an EDTA-containing tube for qPCR analysis. Portions of the liver, spleen, kidney, thymus, and bone marrow were frozen at or below -70°C as backup. FACS analysis, CBC on peripheral blood, and single-cell suspension preparation from spleen and thymus, was performed prior to freezing. Before euthanizing the mice, 200–300 μI of blood was collected via retro-orbital bleed. Blood was divided, processed, and analyzed as described in the Study Specific Necropsy Procedure. Briefly, whole blood was collected in an EDTA-containing tube for complete blood count (CBC) and qPCR analysis. Each animal’s body weight and organs weight were recorded on termination day.

### Leukocyte isolation from thymus, spleen and bone marrow

Single cell suspensions of leukocytes for flow cytometry and qPCR were prepared from portions of thymus (if large enough), spleen and from right femur, tibia, and iliac crests of all mice. Cells were counted by Hemavet (Drew Scientific, FL) and then distributed for assessment by flow cytometry and qPCR. Any remaining cells were cryopreserved for possible later testing.

### Flow cytometry for sub-lineage analysis

Single cell suspensions from thymus (if large enough), spleen, and bone marrow (from sacrificed animals) were prepared and cells were counted using a hemacytometer or automated cell counter. A subset of cells (approximately 1 x 10^6^) was used for flow cytometric analyses. In addition, peripheral blood samples were also analyzed by Flow Cytometry. Surface markers used for immunophenotyping included CD45.1, CD45.2, CD 11b, CD8, CD3, CD4, B220 and Gr-I (Antibodies used: CD45.1-APC, CD45.2-PE, CD 11b-PECy7, CD3-FITC and B220-APC-Cy7, CD4-PE-Cy7, CD8a-APC-Cy7, and Gr-1-APC-cy7all from BD Biosciences, San Jose, CA; cat.no. 561873, 560695, 552850, 555274, 552094, 563933, 557654, and 560600, respectively).

### Histopathology

Multiple tissues were examined for histopathology on all mice in all groups. Liver and gall bladder, kidneys, spleen, thymus, lung, bone marrow (pooled from the right femur, tibia, and iliac crest), heart, and lymph nodes were harvested and fixed in 10% neutral buffered formalin. Fixed tissues were trimmed, embedded in paraffin, sectioned, and stained with Hematoxylin and Eosin. The slides were examined and reported by a board-certified pathologist. The pathologist was blinded to the study groups. Findings in each animal were given a severity score of 0 to 4 (not present, mild lymphoproliferation, moderate lymphoproliferation, severe inflammation and/or lymphoma/tumor present). A histology score of 3 indicated non-malignant mononuclear/lymphoid infiltrates and histology score of 4 indicated a hematologic malignancy.

### Vector Copy Number (VCN)

DNA was extracted from leukocytes isolated from the thymus (or from a thymus tissue piece), spleen, bone marrow, and peripheral blood. DNA isolation and quantification were performed using nanodrop. Quantitative real-time PCR (qPCR) analyses for the number of vector copies per cell (VCN) in tissues were conducted according to the previously published method for detecting wPRE sequence in SFFV RV animals or detecting lentiviral RU5 sequence in G^b^G^M^ and Mock animals [18]. Both negative and positive controls for each vector were included in the qPCR analysis.

### Liver in situ hybridization

The study pathologist reviewed the tissue sections of animal #6 and the decision was made to pursue analysis of this mouse’s liver, where there was lymphoma cell infiltration which was clearly separable from normal hepatocytes. In situ hybridization was conducted on the liver tissue using a probe specific to human gamma globin vector and a scramble control. Formalin-fixed paraffin embedded. Livers were subjected to in situ hybridization, using Digoxigenin labelled Probes (Integrated DNA Technologies, Coralville, IA) using an automated Discovery XT system (Ventana Medical Systems, AZ), according to manufacturer’s protocol. Rabbit anti-digoxin antibody (Sigma-Aldrich, MO) and ChromoMab Blue kit (Ventana Medical Systems) were used to colorize the signals, and the slides were counterstained with Nuclear Fast Red (Polyscientific, NY). The gamma globin probe and a scrambled control were used to probe the liver sections to determine if vector copies were present in the tumor tissue or not. The sequence of the probes were:

Human Gamma Globin Vector Probe Sequence:

[5’-TGCCACCT AGCTGTCCAGGGGTGCCTT AAAATDGCAAACAAGGTITGTTITCTTITCCT-3 ’]

Scramble control Probe Sequence:

Sequence: GTGTAACACGTCTATACGCCCA, # 300514–01, EXIQON

### Statistical analyses

Summary and statistical analyses were based on evaluable animals on all quantitative outcomes including body weight, organ weight, VCN, complete blood counts, and immunophenotyping. To assess if there was any significant difference between treatment groups, one-way ANOVA followed by group-wise comparison with Tukey-Kramer adjustment for multiple testing was used where applicable. For all statistical investigations, tests for significance were two-tailed. A p-value less than the 0.05 significance level was considered to be statistically significant.

## Results

### Efficient transduction and engraftment in primary recipient mice

**Fig 1:**
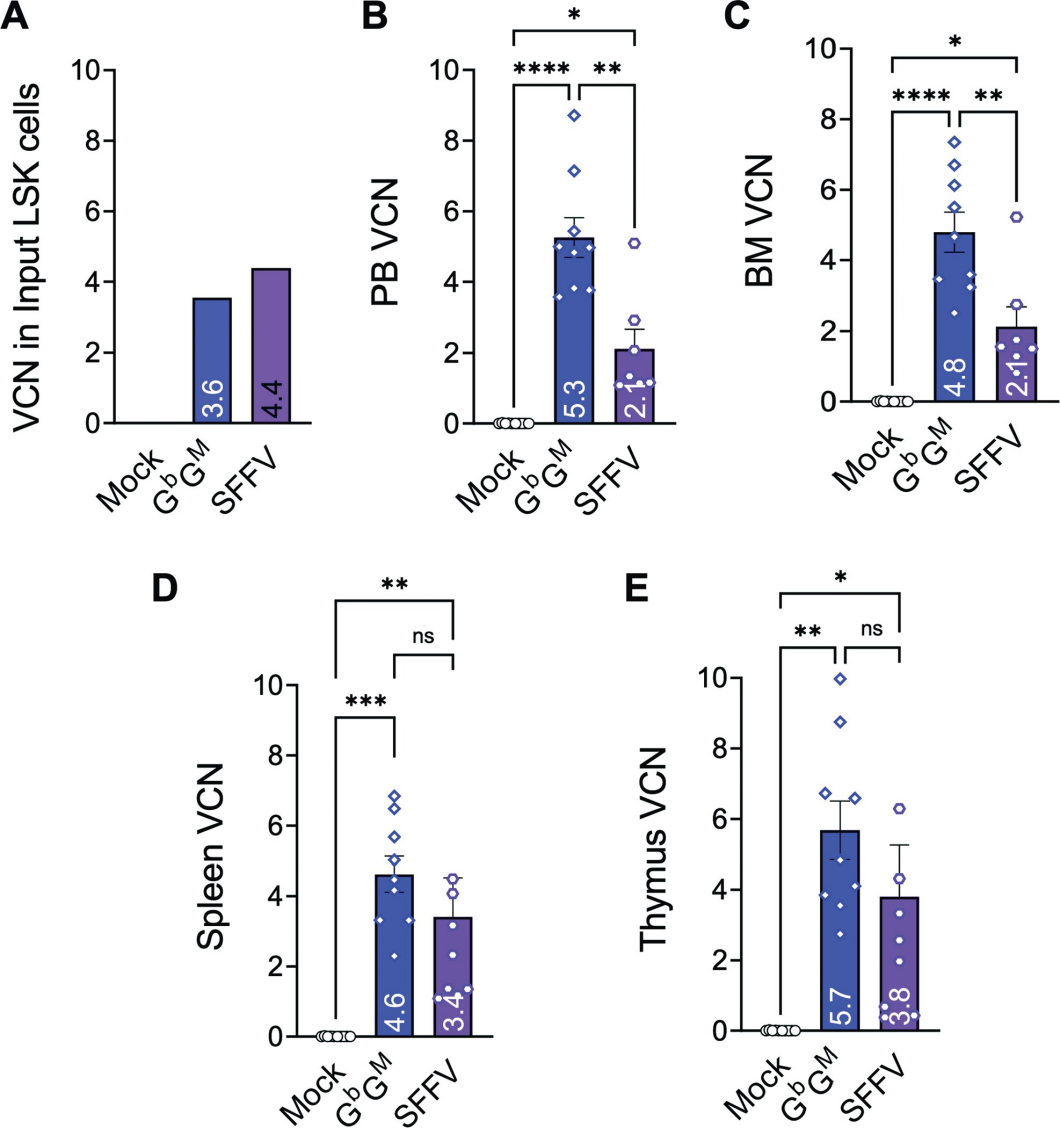
Vector copy number (VCN) analysis after G^b^G^M^ lentiviral (LV) vector and SFFV γ-retro viral (RV) vector gene transduction and transplantation in primary wild-type BoyJ mice. **(A)** Portion of vector transduced mouse hematopoietic stem and progenitor cells (LSK) from C57BL/6 mice (CD45.2) were expanded in culture for 14–16 days to measure the VCN in the input injected LSK cells. Mock transduced cells (no vector) were used as negative control whereas SFFV RV transduced cells are used as positive control (B-E) Transduced LSK cells were transplanted into sub-lethally irradiated BoyJ recipient mice. The VCN in **(B)** PB, **(C)** BM, **(D)** spleen, and **(E)** thymus (analyzed via qPCR) at 8 months after transplant in primary BoyJ recipients are shown. Mean ± standard error of the mean (SEM) is shown for each bar. Each symbol in the bar graph represents an individual animal. Statistics: One-way ANOVA; not significant (ns), *P ≤ .05; **P ≤ .01; ***P ≤ .001, ****P ≤ .0001.

The VCN for the G^b^G^M^ and SFFV RV in the infused LSK cells were 3.5 and 4.4, respectively (**Fig 1A**). As expected, no vector DNA was detected in the Mock group samples. After transplantation, the engraftment was evaluated at week 7 and week 17 in peripheral blood (PB) leukocytes (**S1 Table**). PB engraftment (CD45.2+ donor leukocytes) in the recipient mice for all the groups was ~91% at week 7 and increased to 96% by week 17. The primary recipient mice were followed for 8 months, after which animals were sacrificed and a portion of harvested BM cells were used for VCN and engraftment analysis while the rest were used for secondary transplant. In addition to the BM, PB, spleen, and thymus were collected and both the VCN and engraftment efficiency were evaluated. The terminal mean VCN for G^b^G^M^ group was 5.3±1.69, 4.8±1.70, 4.6±1.53, and 5.7±2.49 in PB, BM, spleen, and thymus, respectively. The latter VCN values were significantly higher than mean VCN for the SFFV RV group in PB (2.1±1.47 versus 5.3±1.69) and BM (2.1±1.49 versus 4.8±1.70) , but not spleen (4.6±1.53 versus 3.6±3.84) or thymus (5.7±2.49 versus 4.0±4.91) (**Fig 1B–1E)**. As expected, no vector copies were detected in any of the Mock-treated groups.

**Fig 2:**
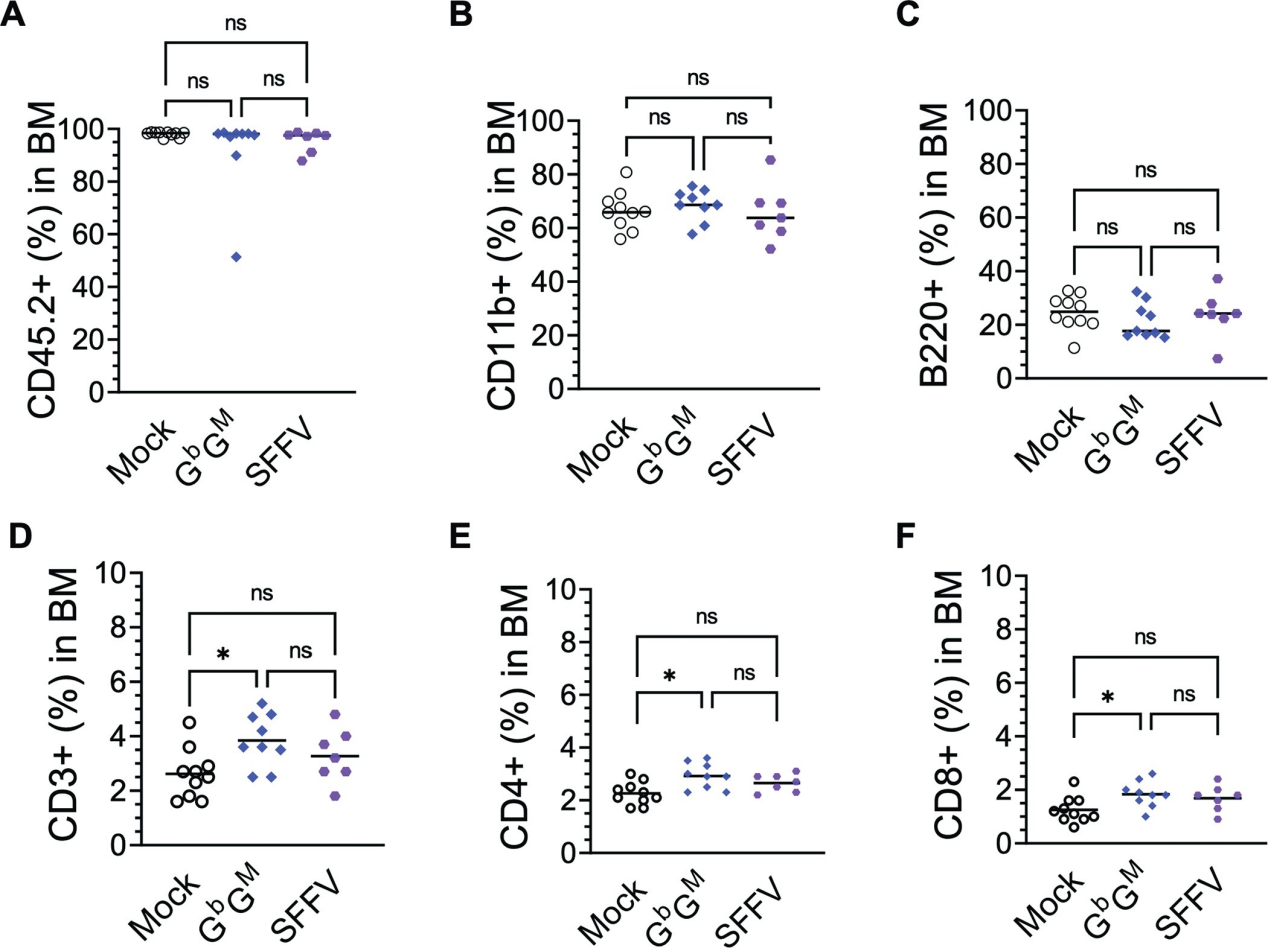
Analysis of engraftment and cell differentiation of the Mock, G^b^G^M^ LV and SFFV RV transduced cells in the BM of primary recipient BoyJ mice. Isolated hematopoietic stem and progenitor (LSK) cells were transduced using G^b^G^M^ LV, SFFV RV and Mock (no vector) and transplanted into primary recipient BoyJ mice. At 8 months after transplant, bone marrow (BM) cells from the mice were collected and analyzed for hematological reconstitution by flow cytometry. **(A)** Donor (CD45.2^+^) cell engraftment in Mock, G^b^G^M^ LV, and SFFV RV mice**. (B-F)** Percentages of myeloid and lymphoid (B and T cell) progeny were evaluated by using antibodies against CD11b, B220, CD3, CD4, and CD8 in Mock, G^b^G^M^ LV, and SFFV RV mice. Each symbol represents an individual animal. Statistics: One-way ANOVA; not significant (ns), *P ≤ .05; **P ≤ .01; ***P ≤ .001, ****P ≤ .0001.

The mean donor cell (CD45.2) engraftment in the BM of the host BoyJ (CD45.1^+^) mice for the Mock, G^b^G^M^, and SFFV RV groups was similar, and greater than 90% (**Fig 2A**). Flow cytometry analysis of BM showed comparable proportions of donor (CD45.2^+^), B cells (CD19^+^ B220+), T cells (CD3^+^ and their subtypes CD4^+^ and CD8^+^ cells), and myeloid cells (CD11b^+^) in all groups (**Fig 2B–2F**). The qualitative and quantitative similarity in engraftment and lineage output in Mock versus SFFV RV and G^b^G^M^ LV groups suggests no alteration of HSC potential following transduction.

Additionally, the persistence of significant VCN in PB and other hematopoietic tissues and the high level of engraftment for 8 months demonstrate that the study animals contained sufficient gene modified cells to provide meaningful assessment of safety.

There were no significant differences in body weight gain and final body weight over the study period in either male or female mice in the primary recipient mice across all experimental groups. At the end of the study, the mean weight for the male Mock group was 30±0.8 g and the mean weight for male G^b^G^M^ treated group was 29±1.6 g. Similarly, the mean weights in female mice were not different between all groups (25±0.4g and 26±1.2g for the Mock and G^b^G^M^ treated groups respectively) **(S1 Fig)**. In addition, no significant difference in absolute organ weights was observed between the three treated groups **S2 Table**. It is worth noting that two animals died within 4 weeks of the primary transplant procedure: one mouse from the G^b^G^M^ group and the other mouse from the SFFV RV group due to the transplant procedure.

### Efficient transduction and engraftment in secondary recipient mice

To assess safety after a second round of HSC regenerative stress, BM cells were isolated from each primary recipient mouse group and were injected into sub-lethally irradiated secondary BoyJ recipient mice in a 1:2 ratio of primary:secondary recipient mice. Analysis of peripheral blood of CD45.2^+^ cells in week 7 following secondary transplant showed high mean PB engraftment of 91.1%, 79.3%, and 87.0% in the Mock, G^b^G^M^, and SFFV RV groups, respectively. PCR analysis showed VCN of 0, 5.1, and 2.0 for the Mock, G^b^G^M^, and SFFV RV groups, respectively (**Table 1)**.

**Table 1:** Transplant details and engraftment after secondary transplantation.

Time	Primary Recipients	Mock	G^b^G^M^	SFFV
**Predose**	# of mice	20	18	14
	# of LSK cells	10–25 x 10^6^		
**Week 7**	# of evaluable mice	20	17	14
	Mean PB VCN	0	5.1	2.0
	PB engraftment (%)	91.1	79.3	87.0
**Week 17**	# of evaluable mice	20	15	14
	Mean PB VCN	0	5.6	2.2
	PB engraftment (%)	96.0	85.0	93.6

Abbreviations: Mock = mice transplanted with untransduced bone marrow cells; G^b^G^M^ = recombinant γ-globin lentivirus vector with point mutation; SFFV = spleen focus-forming virus based γ-retrovirus, PB = peripheral blood, VCN = vector copy number Mice with donor cells engraftment ≥30% and VCN ≥ 0.2 as determined at the early 7-week time point were considered evaluable at the end of the study.

As mentioned earlier, only the mice that met the study criteria were evaluated at the end of the study. The numbers of mice that met the study criteria were 20, 15, and 14 mice for the Mock, G^b^G^M^, and SFFV RV groups, respectively. At week 17 post-transplant, the mean PB engraftment was 96.0%, 85.0%, and 93.6% for the Mock, G^b^G^M^, and SFFV RV groups, respectively (**Table 1**). PCR analysis showed VCN of 0, 5.6, and 2.2 for the Mock, G^b^G^M^, and SFFV RV groups, respectively (**Table 1).** The VCN at week 17 was stable compared to the earlier timepoint of 7 weeks, demonstrating stable engraftment of transduced HSC.

**Fig 3:**
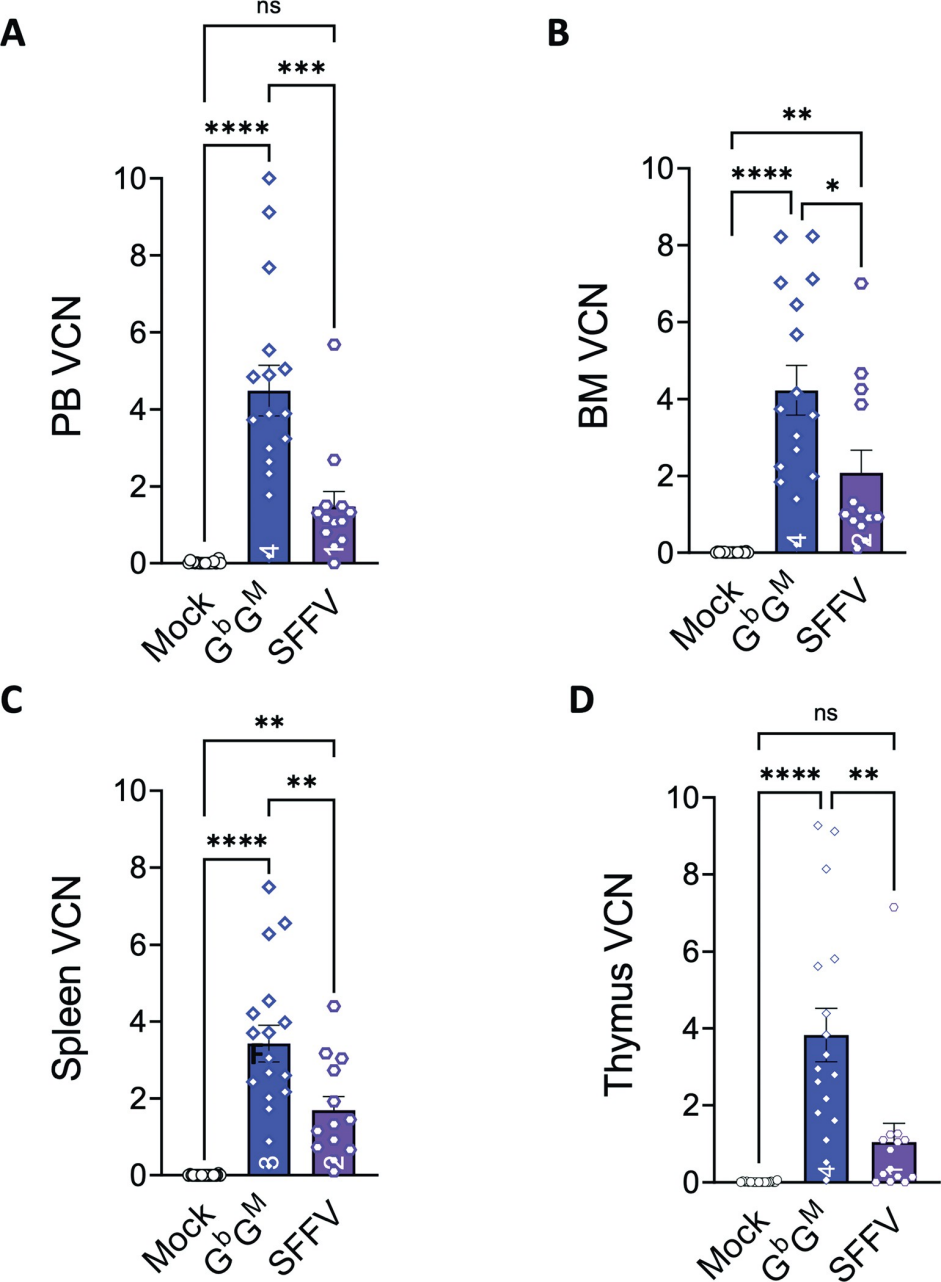
Vector copy number (VCN) analysis after G^b^G^M^ LV and SFFV RV animals in secondary transplanted BoyJ mice. Isolated bone marrow cells from the primary recipient BoyJ mice were processed and transplanted into sub-lethally irradiated secondary recipient BoyJ mice to assess the HSC engraftment. The vector copy number (VCN) in **(A)** peripheral blood (PB), **(B)** bone marrow (BM), **(C)** spleen, and **(D)** thymus was analyzed via qPCR at 10 months after transplant in secondary recipients is shown. Mean and standard error of the mean are shown for each bar. Each symbol in the bar graph represents an individual animal. Statistics: One-way ANOVA; not significant (ns), *P ≤ .05; **P ≤ .01; ***P ≤ .001, ****P ≤ .0001.

At the terminal sacrifice (10 months post-transplant), PB, BM, spleen and thymus of the secondary mice were collected, and both VCN and engraftment were evaluated. At experiment termination, the mean VCN for the G^b^G^M^ group was 4.5±2.62, 4.2±2.56, 3.4±1.97, and 3.8±2.87 in PB, BM, spleen, and thymus, respectively. These VCN values were significantly higher than the mean VCN values for the SFFV group (1.47±1.42, 2.1±2.03, 1.7±1.28, and 1.04±1.83 in PB, BM, spleen, and thymus) (**Fig 3A–3D)**. As expected, no vector copies were detected in any of the Mock-treated groups.

**Fig 4:**
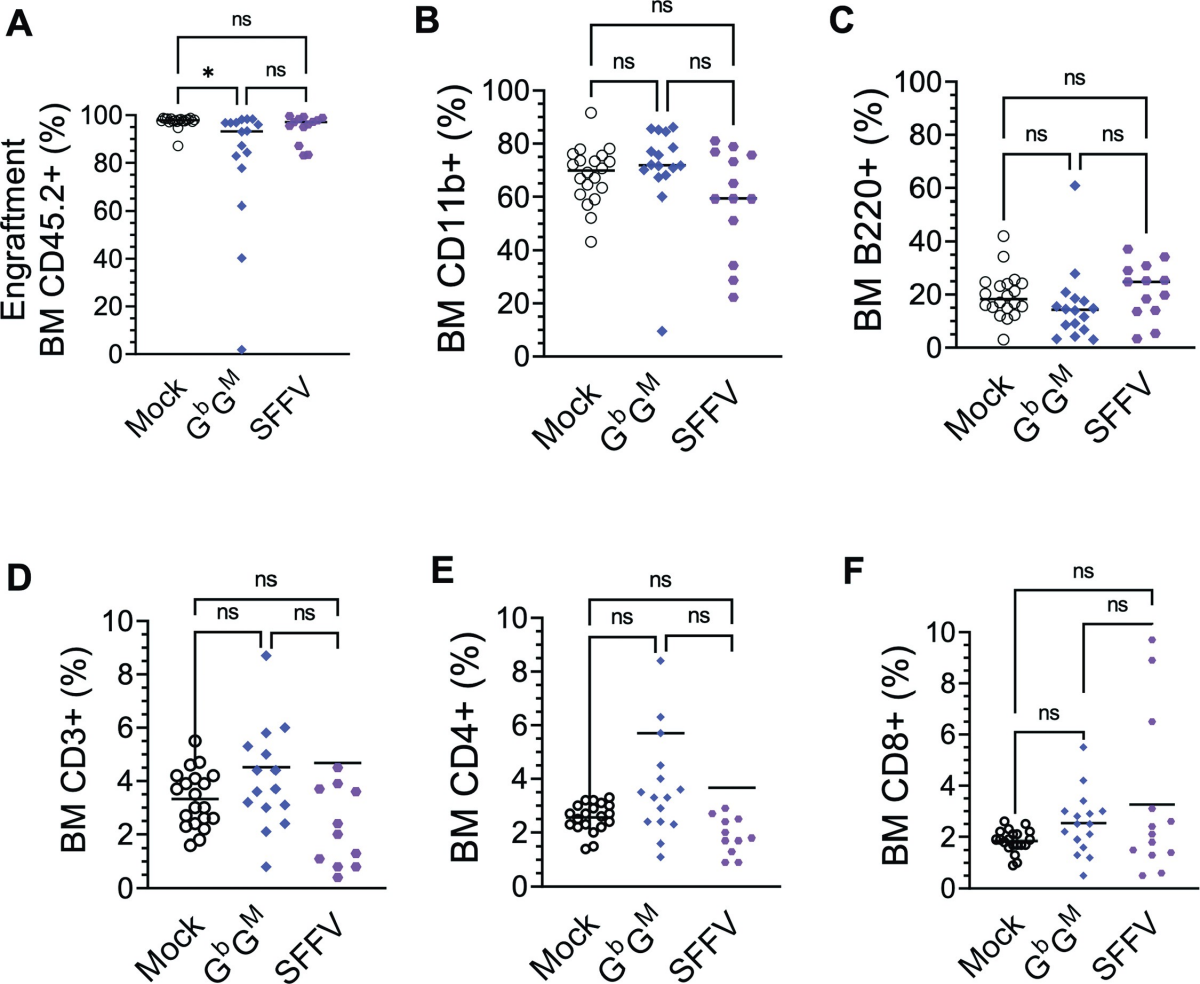
Analysis of engraftment and cell differentiation of the Mock, G^b^G^M^ LV and SFFV RV transduced cells in the BM of Secondary BoyJ recipient mice. Isolated BM cells from primary mice were processed and transplanted in secondary recipient BoyJ mice. (A) Donor mouse (CD45.2+) cell engraftment in Mock, G^b^G^M^, and SFFV RV secondary mice 10 months after transplant. **(B-F)** Percentages of myeloid and lymphoid (B and T) cells were evaluated by using antibodies against CD11b, B220, CD3, CD4, and CD8 in Mock, G^b^G^M^, and SFFV mice. Each symbol represents an individual animal. Statistics: One-way ANOVA. not significant (ns), *P ≤ .05; **P ≤ .01; ***P ≤ .001, ****P ≤ .0001.

The long-term persistence of a stable VCN and engraftment in primary mice for 8 months and then for an additional 10 months in secondary mice demonstrates the efficient transduction and stable engraftment of the transduced hematopoietic stem cells (HSC), and that the study animals had sufficient genetically modified HSCs to provide meaningful safety assessment. The bone marrow engraftment of the transplanted CD45.2^+^ donor cells and the sub-lineage analysis were comparable among the three groups **(Fig 4)**. Flow cytometry analysis of bone marrow showed comparable proportions of donor (CD45.2^+^) B cells (CD19^+^ and B220^+^ cells), T cells (CD3+and its subtypes CD4^+^ and CD8^+^), and myeloid cells (CD11b^+^) (**Fig 4B–4F**). The qualitative and quantitative similarity in engraftment and lineage output in secondary SFFV RV and G^b^G^M^ LV mice, compared to Mock secondary control mice, are suggestive of unaltered HSC potential through the transduction, primary and secondary transplants.

There were no significant changes in the body weight of both male and female G^b^G^M^ mice over the entire study period (**S1C and S1D Fig**). Two animals died in the G^b^G^M^ treated group; one unscheduled death took place right after the 7-week time-point after a blood collection procedure and the second animal was euthanized due to a festering wound.

In general, no statistically significant differences in organ weight between the Mock and G^b^G^M^-treated groups were noted. For the SFFV RV treated group, no statistically significant differences between the Mock and SFFV RV treated groups were noted in all tissues, except for the weight of spleen. The mean spleen weight harvested from the terminal endpoint of the secondary transplant for the Mock, G^b^G^M^, and SFFV RV-treated groups was 0.11±0.047g, 0.14±0.115g, and 0.20±0.165g respectively (**S3 Table**). A significant increase in the mean spleen weight was observed in the SFFV RV as compared to the Mock control group (*p*<0.005).

### Hematological findings after treatment in G^b^G^M^ and SFFV RV treated mice

PB samples were collected 8 and 10 months post primary and secondary transplants, respectively. No significant changes were observed in the hematological parameters attributable to the administration of G^b^G^M^ compared to the Mock group (**S4A Table).** Comparison between the mean values of the Mock and G^b^G^M^ experimental groups did not show any statistical differences between the primary and secondary recipients. On the other hand, the secondary recipient mice that were initially treated with the SFFV showed a significant increase in the white blood cells count when compared to the Mock or the primary recipient SFFV RV group. The white blood cell parameters, such as neutrophils, lymphocytes, monocytes, eosinophils, and basophils, were increased by 3-50-fold in the SFFV RV group compared to Mock or the primary recipient SFFV RV group (**S4B Table)**.

### Histopathology and development of hematological malignancy

Tissues were harvested at 8 months and 10 months post-transplant from the primary and secondary animals, respectively, and evaluated by a board-certified pathologist. Tissues from the primary and secondary recipient mice were evaluated for inflammation, hematological neoplasm, and non-hematological neoplasm. In all groups, including the Mock group, mild inflammation findings were observed in the lung and liver, which could be attributed to the irradiation and/or transplant process. In the primary and secondary recipient mice, no abnormal findings (score 0) were observed in thymus, lymph nodes, and heart in all the groups. In addition, no abnormal inflammation findings were observed in the BM and spleen of all the groups. There was mild to moderate inflammation of the kidney in two animals, but the findings were similar to those observed in the Mock treated group with inflammatory mononuclear/lymphoid infiltrate.

One of the safety endpoints of this study was to evaluate the development of vector positive hematological malignancies in the treated animals after the primary and secondary transplants. In the first transplant cohort, one out of nine mice in the G^b^G^M^ had non-malignant mononuclear/lymphoid infiltrates (score 3) (**Table 2**). On the other hand, two out of nine mice in the SFFV-treated group showed either non-malignant mononuclear/lymphoid infiltrates or hematologic malignancy (score 4). In the secondary transplant cohort, three out of twenty mice in the Mock group had score 3, whereas three out of seventeen mice in the G^b^G^M^ group had score range between 3 to 4 (**Table 2).**

**Table 2:** Animals with lymphoproliferation or malignancy.

Group	Sex	Primary			Secondary		
		Evaluable	Score 3^a^	Score 4^b^	Evaluable	Score 3^a^	Score 4^b^
Mock	Male	5	0	0	10	0	0
	Female	5	0	0	10	3	0
G^b^G^M^	Male	5	0	0	9	0	1
	Female	4	1	0	8	1	1
SFFV	Male	4	1	0	6	1	3
	Female	5	0	1	8	0	0

Abbreviations: Mock = mice transplanted with un-transduced bone marrow cells; G^b^G^M^ = recombinant γ-globin lentivirus vector with point mutation in the γ-globin gene; SFFV = spleen focus-forming virus. a A histology score of 3 indicates non-malignant mononuclear/lymphoid infiltrates. b A histology score of 4 indicates a hematologic malignancy.

A total of four mice in the SFFV RV group showed donor-derived vector-positive lymphomas/ leukemias (score 4): one animal in the primary transplant group and three mice in the secondary transplant group. Mice with a malignancy present in any organ were closely investigated, and FACS analysis performed on tumor cells for host (CD45.1) versus donor cell markers (CD45.2) and lineage markers. Tumor tissues were tested for presence of the vector by qPCR. Details of mice with donor cell-derived hematological malignancies are listed in **Table 3**.

**Table 3:** Animals with donor-derived and host-derived malignancies.

Animal ID	Group	Scheduled Sacrifice	Tissues Involved	VCN (copies/cell), (% donor)	Type of Malignancy	Origin	Pathological Interpretation
**1**	SFFV#	No	BM, spleen, lung, liver, kidney, heart	Spleen 13.25 Thymus 16.00 Lung 15.64	Lymphoma	Donor-derived, vector-positive	Tumour negative for CD3, not a T-cell lymphoma.
**2**	SFFV*	No	BM, lung, kidney	Thymus 0.21 Lung 4.09 Kidney 0.99	Lymphoma	Donor-derived, vector-positive	Tumour negative for CD3 and CD79a, so possibly histiocytic in origin.
**3**	SFFV*	Yes	BM, spleen, LN, lung, liver, kidney, heart	BM 1.00 (94.2%) Blood 1.33 (73.4%) Spleen 1.33 (72%) Thymus 1.24 (91.3%)	Lymphoma	Donor-derived vector-positive	IHC negative for CD3, CD68, and CD79a, tumour could be further classified based on IHC.
**4**	SFFV*	Yes	BM, spleen, lung, liver, kidney	BM 1.12 (89.2%) Blood 1.50 (66.8%) Spleen 1.92 (82.9%) Thymus 1.09 (97.7%)	Leukemia	Donor-derived, vector-positive	Highly cellular with significantly higher population of neutrophils (myeloid to erythroid ratio 15:1). B-Myeloid
**5**	G^b^G^M^*	No	BM, spleen, lung, liver, kidney	BM 0.21 (1.9%) Blood 0.19 (1.5%) Spleen 0.24 (1.9%) Thymus 0.06 (0.1%)	Lymphoma	Host-derived, vector-negative	Neoplasm is negative for CD3 and CD79a. CD68 staining not performed. Further classification of lymphoma was not possible.
**6**	G^b^G^M^*	Yes	BM, spleen, lung, liver, kidney	BM 2.24 (32.8%) Blood 3.73 (29.8%) Spleen 3.05 (9.7%) Thymus 0.51 (3.5%)	Lymphoma	Host-derived	Neoplasm is confirmed to be T-cell lymphoma by CD3 staining.

BM = Bone marrow; LN = lymph node; IHC = immunohistochemistry.

#Primary mice

*Secondary mice.

Two of the SFFV RV mice with lymphomas were found dead. Animal 1 was found dead 4.5 months post-primary transplant. Lymphoma was observed in multiple tissues, including the spleen, lung, kidney, and heart. FACS analysis showed that the tumor was donor cell-derived, and the VCN was high, 12 and 14 in the spleen and thymus, respectively, indicative of donor-derived CD45.2+ cells, likely vector-induced hematological malignancy. The tumor was negative for CD3^+^ cells, ruling out T-cell lymphoma. Similar observations were made for Animal 2, which was found dead at 8.6 months post-secondary transplant. Here, the tumor was also donor-derived, vector positive, but negative for T and B cell markers and was diagnosed likely to be of histiocytic origin (**Table 3**). Similarly, FACS and VCN data from animals 3 and 4 showed that the tumor was donor cell-derived and vector-positive, but lineage markers tested were not detected. Hence, the lymphoma type remained unclassified. Both animals had tumors had VCN >1. Animal 4 in the SFFV RV group showed that tissues were highly cellular with a significant population of neutrophils. These findings were confirmed by FACS analysis with unusually high percentage of CD11b^+^/Gr-1^+^ cells in spleen. In addition, high percentages of CD11b^+^/B220^+^ cells in BM and spleen were observed, indicating a potential B-myeloid leukemia.

Two G^b^G^M^-treated animals developed malignant or premalignant lymphoproliferation that originated from host cells (CD45.1+) and were therefore not the result of G^b^G^M^ treatment.

Animal 5 was found dead 6 months post-secondary transplant. PCR and FACS analysis of the tumor yielded low VCN (<0.2) and low levels of donor CD45.2+ cells (<2%), and the tumor tissue was of host (CD45.1+) origin. In Animal 6 FACS analysis of the spleen showed that >50% of the cells in the spleen were host-derived cells (CD45.1^+^) that were abnormal lymphoma cells, and 6% were donor-derived leukocytes (CD45.2^+^). The rest were erythroid cells (CD45-), which could be from the host or donor, as erythroid cells do not express any isoform of CD45 and are negative for both markers (**S2 Fig**). This animal had relatively high VCN in blood, bone marrow, and spleen, and low VCN in thymus despite having a CD45.1+ (host derived) lymphoma. Approximately 12–13% of the splenocytes were of donor origin (CD45.2^+^) with representatives of myeloid, T-cells, and B-cells with a typical distribution of cells within the spleen. However, the majority of the splenocytes were host-derived cells that were CD3^-^CD4^+^, with some double positive for CD4 and CD8, which are atypical T-cells. This abnormal distribution of lineage markers of host origin demonstrates that the tumor is a T cell lymphoma of host origin, with VCN contribution from erythroid cells transduced with G^b^G^M^. Details of the FACS analysis of this animal are shown in **S2 Fig,** suggesting that the lymphoma cells were possibly host (CD45.1^+^) T cell- derived. However, the relatively high VCN in this mouse warrants further investigation to reconcile the two observations.

### Liver in situ hybridization

**Fig 5:**
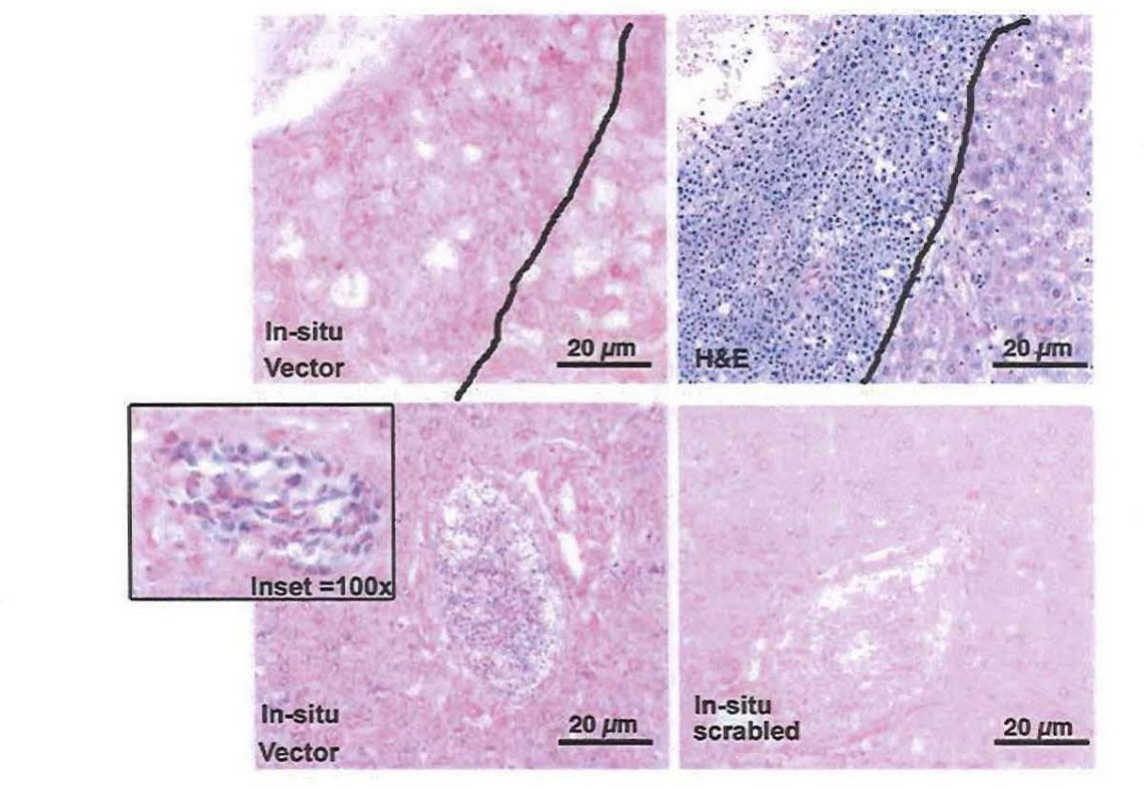
Liver histopathology and in situ hybridization revealing the origin of lymphoma. Liver sample from mouse #6 in the GbGM mice group showed a demarcated lymphoma tumor and normal tissue was obtained (H&E stain). The in situ hybridization analysis using the gamma globin vector showed absence of vector sequence in the tumor/lymphoma tissue. However, the in situ analysis showed the presence of vector in hematopoietic cells in the blood vessel and in the liver sinusoidal vessels distributed throughout the liver (100x). The scrambled probe did not show any abnormal staining in the liver tissue.

To further investigate the origin of lymphoma, in situ hybridization on the liver tissue obtained from one of the G^b^G^M^ mice (mouse #6) was investigated using a probe specific to human gamma globin vector and scramble control. The liver from mouse #6 was obtained, and H&E staining clearly showed the region of demarcated lymphoma tumor and normal tissue (Fig 5). The in situ hybridization analysis using the gamma globin vector showed absence of vector sequence in the tumor/lymphoma tissue. However, the in situ analysis showed the presence of vector in hematopoietic cells in the blood vessel and in the liver sinusoidal vessels distributed throughout the liver. As expected, the scrambled probe did not show any abnormal staining in the liver tissue. These results clearly suggest that animal #6 has a host-derived lymphoma that is gamma globin vector negative.

## Discussion

Autologous HSPC transduced *ex vivo* with an LV represents a promising approach to treat monogenic disorders such as SCA [21]. However, one needs to rigorously rule out the risk of hematological malignancy, which is dependent on the vector design. The risk is expected to be negligible with an LV that carries erythroid-specific promoter/enhancer elements. Herein, we demonstrated the preclinical safety of a modified γ-globin LV, G^b^G^M^, in mice that underwent primary and secondary transplants. No donor-derived hematological malignancies were detected in any of the G^b^G^M^-treated mice after primary and secondary BMT. On the other hand, and as reported previously, mice treated with the SFFV RV developed donor-derived malignancies.

For the G^b^G^M^ LV safety assessment, wild-type mice were chosen instead of SCD mouse models to avoid any potential disease-related complications in the disease model, which could be severe in the Mock and SFFV groups, confounding the study. In the present model, no significant variabilities were observed between the Mock, SFFV RV, and G^b^G^M^ groups with regards to body weight, donor cell engraftment, or the differentiation of donor hematopoietic cells to sub-lineages. Interestingly, similar engraftment efficiencies were observed between the primary and secondary recipient mice **(Figs 2 and 4)**, which demonstrated that the treated groups were comparable to the non-transduced Mock HSCs. All primary and secondary recipients demonstrated more than 90% BM engraftment of genetically manipulated HSPC with clinically relevant VCN, demonstrating robust gene transfer into HSC and providing meaningful safety results. Engrafted cells proliferated and differentiated into various functional cells, such as B cells, T cells, and myeloid cells. The absence of any major engraftment variabilities among all the groups in secondary transplanted mice emphasizes that the study conditions are adequate to assess the safety of G^b^G^M^ transplanted cells. However, there were differences in the measured in vivo VCN, white blood cell counts, spleen weight, and histopathology findings between the treated groups, as discussed below.

There were significant differences in the VCN levels between the G^b^G^M^ and SFFV-treated groups. Interestingly, the G^b^G^M^ LV group exhibited at least two-fold greater VCN than the SFFV RV-treated group. This could be due to the fact LV is known to transduce both mitotic and post-mitotic cells, whereas RV transduces only mitotic cells [22]. Since RV transduction protocol requires cytokine pre-stimulation of HSC to force them to divide, a significant proportion of HSCs lose their stem cell potential. This is likely the reason for the high input VCN of SFFV RV HSPC versus a lower VCN *in vivo*. VCN of the G^b^G^M^ LV group was maintained and even higher in vivo probably because of the short transduction period, not requiring HSC division, thereby preserving HSC ‘stemness’. It has been reported that there is a direct relationship between VCN and the risk of IO [23]. However, despite the fact that VCN in BM, PB, spleen, and thymus in the G^b^G^M^ test group were higher than those in the SFFV RV group for both the primary and secondary recipients, no donor-derived vector-induced malignancies were observed in the G^b^G^M^ treated group. In contrast, 4 SFFV RV mice showed donor-derived vector-positive lymphomas despite nearly half the VCN/vector insertions, further bolstering the safety of the G^b^G^M^ LV.

Although the primary recipient mice did not show differences in spleen weight and white blood cell counts in this study, the secondary recipient SFFV RV group showed statistical increases in both parameters compared to the Mock and G^b^G^M^ groups. This increase in spleen weight and white blood cell counts in the secondary recipient SFFV RV group is consistent with clinical findings related to rapid increase in white blood cells and the infiltration of malignant cells into the spleen leading to splenomegaly in hematologic malignancies, such as lymphomas, leukemias, myeloproliferative disorders [24–26].

The primary goal of this study was to evaluate the potential of developing hematological malignancies (such as leukemia or lymphoma) after the treatment of G^b^G^M^ and long-term follow-up. A total of six animals developed hematological malignancies during this study. Four of the six mice were in animals treated with the SFFV RV. Thorough analysis revealed that the tumors were donor-derived (CD45.2+) and vector-positive malignancies. SFFV RV has been reported to cause leukemia by insertional up-regulation of proto-oncogenes expression in mice and in clinical trials of Wiscott-Aldrich Syndrome and chronic granulomatous disease [24–26]. Therefore, the development of lymphoma or leukemia in the SFFV RV-treated groups aligns well with previous reports [20], and validates our safety study. On the other hand, two mice in the G^b^G^M^ group developed host cell-derived malignancy. Host cell-derived lymphomas have been shown to occur at a frequency of 2–24% using this mouse model [20,27,28], and are likely due to age and irradiation-induced for the transplant and rule out any connection to the G^b^G^M^ treatment. One of the two mice had the presence of the vector due to the high percentage of normal erythroid cells that were negative for either host or donor CD45 markers in the infiltrated organs. This animal was analyzed in depth twice, once using fresh cells, when sacrificed, and again on a cryopreserved vial of cells. Both times, the FACS analysis clearly showed a host derived CD4+ T cell lymphoma and the few donor-derived cells present showed the appropriate normal percentages of CD3+, CD4+ and CD8+ cells, and presence of a significant proportion of CD45- erythroid cells. The in situ hybridization experiment demonstrates that while the vector was present in the hematopoietic cells in this animal in non-tumor bearing areas, the tumor tissue infiltrating the liver was negative for the vector (Fig 5). Furthermore, the flow cytometry analysis showed that the tumor was of host (CD45.2) origin (S2 Fig). Hence, the high vector copy numbers in the spleen were probably derived from the residual transduced donor leukocytes (CD45.1+) and donor erythroid cells (CD45 negative) in the spleen. This indicates that animal#6 has a host-derived lymphoma that is vector negative.

In conclusion, the study protocol was appropriate for detecting the development of hematological malignancies driven by insertional mutagenesis with the G^b^ G^M^ LV. Most importantly, the study demonstrated not only the high engraftment efficacy, but also the safety of G^b^G^M^ after primary and secondary BMT. The LV had no adverse long-term effects on transduced mouse HSC and their hematopoietic progeny.

## Supporting information

S1 Fig Longitudinal total body weight of male and female mice from the mock, G^b^G^M^, and SFFV treated group after primary and secondary BM transplants. (PDF)

S2 Fig Engraftment and multi-lineage reconstitution in spleen of one lymphoma bearing secondary recipient mice. (PDF)

S1 Table Transplant and engraftment after primary transplantation. (PDF)

S2 Table Mean organ weights of the primary transplanted mice with Mock, GbGM, and SFFV at 8 months post-transplant. (PDF)

S3 Table Mean organ weights of the secondary transplanted mice with Mock, GbGM, and SFFV at 10 months post-transplant. (PDF)

S4 Table A. Hematological analyses of WT BoyJ recipient mice following the transplant with GbGM, Mock, and SFFV transduced HSPC 8 months following primary transplant. B. Hematology analyses of secondary WT BoyJ recipient mice following the injection of BM of primary mice transplanted with GbGM, mock, and SFFV transduced HSPCs 10 months post-secondary transplant.
(PDF)

## References

[1] Sundd P, Gladwin MT, Novelli EM. Pathophysiology of Sickle Cell Disease. Annu Rev Pathol. 2019;14:263–92. Epub 20181017. doi: 10.1146/annurev-pathmechdis-012418-012838 ; PubMed Central PMCID: PMC7053558. 30332562 PMC7053558

[2] Bridges KR, Barabino GD, Brugnara C, Cho MR, Christoph GW, Dover G, et al. A multiparameter analysis of sickle erythrocytes in patients undergoing hydroxyurea therapy. Blood. 1996;88(12):4701–10. 8977264

[3] Robinson TM, Fuchs EJ. Allogeneic stem cell transplantation for sickle cell disease. Curr Opin Hematol. 2016;23(6):524–9. Epub 2016/10/18. doi: 10.1097/MOH.0000000000000282 ; PubMed Central PMCID: PMC5130409. 27496639 PMC5130409

[4] Hsieh MM, Fitzhugh CD, Weitzel RP, Link ME, Coles WA, Zhao X, et al. Nonmyeloablative HLA-matched sibling allogeneic hematopoietic stem cell transplantation for severe sickle cell phenotype. JAMA. 2014;312(1):48–56. Epub 2014/07/25. doi: 10.1001/jama.2014.7192 ; PubMed Central PMCID: PMC4698790. 25058217 PMC4698790

[5] Hsieh MM, Kang EM, Fitzhugh CD, Link MB, Bolan CD, Kurlander R, et al. Allogeneic hematopoietic stem-cell transplantation for sickle cell disease. N Engl J Med. 2009;361(24):2309–17. PubMed doi: 10.1056/NEJMoa0904971 . 20007560 PMC3627532

[6] Kanakry JA, Luznik L. Might haplo "be the (better) match"? Blood. 2016;127(7):799–800. Epub 2016/02/20. doi: 10.1182/blood-2016-01-689042 ; PubMed Central PMCID: PMC4760085 interests. 26893397 PMC4760085

[7] Kohn DB, Booth C, Shaw KL, Xu-Bayford J, Garabedian E, Trevisan V, et al. Autologous Ex Vivo Lentiviral Gene Therapy for Adenosine Deaminase Deficiency. N Engl J Med. 2021;384(21):2002–13. Epub 2021/05/12. doi: 10.1056/NEJMoa2027675 ; PubMed Central PMCID: PMC8240285. 33974366 PMC8240285

[8] Kohn DB, Booth C, Kang EM, Pai SY, Shaw KL, Santilli G, et al. Lentiviral gene therapy for X-linked chronic granulomatous disease. Nat Med. 2020;26(2):200–6. Epub 2020/01/29. doi: 10.1038/s41591-019-0735-5 . 31988463 PMC7115833

[9] Eichler F, Duncan C, Musolino PL, Orchard PJ, De Oliveira S, Thrasher AJ, et al. Hematopoietic Stem-Cell Gene Therapy for Cerebral Adrenoleukodystrophy. N Engl J Med. 2017;377(17):1630–8. Epub 2017/10/05. doi: 10.1056/NEJMoa1700554 ; PubMed Central PMCID: PMC5708849. 28976817 PMC5708849

[10] Thompson AA, Walters MC, Kwiatkowski J, Rasko JEJ, Ribeil JA, Hongeng S, et al. Gene Therapy in Patients with Transfusion-Dependent beta-Thalassemia. N Engl J Med. 2018;378(16):1479–93. Epub 2018/04/19. doi: 10.1056/NEJMoa1705342 . 29669226

[11] Ribeil JA, Hacein-Bey-Abina S, Payen E, Magnani A, Semeraro M, Magrin E, et al. Gene Therapy in a Patient with Sickle Cell Disease. N Engl J Med. 2017;376(9):848–55. doi: 10.1056/NEJMoa1609677 . 28249145

[12] Hacein-Bey Abina S, Gaspar HB, Blondeau J, Caccavelli L, Charrier S, Buckland K, et al. Outcomes following gene therapy in patients with severe Wiskott-Aldrich syndrome. JAMA. 2015;313(15):1550–63. Epub 2015/04/22. doi: 10.1001/jama.2015.3253 ; PubMed Central PMCID: PMC4942841. 25898053 PMC4942841

[13] Kanter J, Thompson AA, Pierciey FJ Jr., Hsieh M, Uchida N, Leboulch P, et al. Lovo-cel gene therapy for sickle cell disease: Treatment process evolution and outcomes in the initial groups of the HGB-206 study. Am J Hematol. 2023;98(1):11–22. Epub 2022/09/27. doi: 10.1002/ajh.26741 . 36161320 PMC10092845

[14] Hacein-Bey-Abina S, Von Kalle C, Schmidt M, McCormack MP, Wulffraat N, Leboulch P, et al. LMO2-associated clonal T cell proliferation in two patients after gene therapy for SCID-X1. Science. 2003;302(5644):415–9. doi: 10.1126/science.1088547 . 14564000

[15] Hacein-Bey-Abina S, Garrigue A, Wang GP, Soulier J, Lim A, Morillon E, et al. Insertional oncogenesis in 4 patients after retrovirus-mediated gene therapy of SCID-X1. J Clin Invest. 2008;118(9):3132–42. PubMed doi: 10.1172/JCI35700 . 18688285 PMC2496963

[16] Modlich U, Navarro S, Zychlinski D, Maetzig T, Knoess S, Brugman MH, et al. Insertional Transformation of Hematopoietic Cells by Self-inactivating Lentiviral and Gammaretroviral Vectors. Mol Ther. 2009. Epub 2009/08/13. doi: 10.1038/mt.2009.179 [pii] 10.1038/mt.2009.179. . 19672245 PMC2835038

[17] Arumugam PI, Higashimoto T, Urbinati F, Modlich U, Nestheide S, Xia P, et al. Genotoxic potential of lineage-specific lentivirus vectors carrying the beta-globin locus control region. Mol Ther. 2009;17(11):1929–37. doi: 10.1038/mt.2009.183 ; PubMed Central PMCID: PMC2835044. 19707188 PMC2835044

[18] Perumbeti A, Higashimoto T, Urbinati F, Franco R, Meiselman HJ, Witte D, et al. A novel human gamma-globin gene vector for genetic correction of sickle cell anemia in a humanized sickle mouse model: critical determinants for successful correction. Blood. 2009;114(6):1174–85. doi: 10.1182/blood-2009-01-201863 ; PubMed Central PMCID: PMC2723013. 19474450 PMC2723013

[19] Kiem HP, Arumugam PI, Burtner CR, Fox CF, Beard BC, Dexheimer P, et al. Pigtailed macaques as a model to study long-term safety of lentivirus vector-mediated gene therapy for hemoglobinopathies. Mol Ther Methods Clin Dev. 2014;1:14055. doi: 10.1038/mtm.2014.55 ; PubMed Central PMCID: PMC4448740. 26052523 PMC4448740

[20] Modlich U, Kustikova OS, Schmidt M, Rudolph C, Meyer J, Li Z, et al. Leukemias following retroviral transfer of multidrug resistance 1 (MDR1) are driven by combinatorial insertional mutagenesis. Blood. 2005;105(11):4235–46. Epub 20050215. doi: 10.1182/blood-2004-11-4535 . 15713797

[21] Abraham AA, Tisdale JF. Gene therapy for sickle cell disease: moving from the bench to the bedside. Blood. 2021;138(11):932–41. doi: 10.1182/blood.2019003776 34232993 PMC9069474

[22] Dufait I, Liechtenstein T, Lanna A, Bricogne C, Laranga R, Padella A, et al. Retroviral and lentiviral vectors for the induction of immunological tolerance. Scientifica (Cairo). 2012;2012. Epub 2013/03/26. doi: 10.6064/2012/694137 ; PubMed Central PMCID: PMC3605697. 23526794 PMC3605697

[23] Uchiyama T, Takahashi S, Nakabayashi K, Okamura K, Edasawa K, Yamada M, et al. Nonconditioned ADA-SCID gene therapy reveals ADA requirement in the hematopoietic system and clonal dominance of vector-marked clones. Mol Ther Methods Clin Dev. 2021;23:424–33. Epub 2021/11/18. doi: 10.1016/j.omtm.2021.10.003 ; PubMed Central PMCID: PMC8566957. 34786435 PMC8566957

[24] Sadat MA, Dirscherl S, Sastry L, Dantzer J, Pech N, Griffin S, et al. Retroviral vector integration in post-transplant hematopoiesis in mice conditioned with either submyeloablative or ablative irradiation. Gene Ther. 2009;16(12):1452–64. PubMed doi: 10.1038/gt.2009.96 . 19657370 PMC2795029

[25] Stein S, Ott MG, Schultze-Strasser S, Jauch A, Burwinkel B, Kinner A, et al. Genomic instability and myelodysplasia with monosomy 7 consequent to EVI1 activation after gene therapy for chronic granulomatous disease. Nat Med. 2010;16(2):198–204. Epub 2010/01/26. doi: 10.1038/nm.2088 . 20098431

[26] Ginn SL, Liao SH, Dane AP, Hu M, Hyman J, Finnie JW, et al. Lymphomagenesis in SCID-X1 mice following lentivirus-mediated phenotype correction independent of insertional mutagenesis and gammac overexpression. Mol Ther. 2010;18(5):965–76. doi: 10.1038/mt.2010.50 ; PubMed Central PMCID: PMC2890120. 20354504 PMC2890120

[27] Will E, Bailey J, Schuesler T, Modlich U, Balcik B, Burzynski B, et al. Importance of murine study design for testing toxicity of retroviral vectors in support of phase I trials. Mol Ther. 2007;15(4):782–91. PubMed doi: 10.1038/sj.mt.6300083 . 17299409

[28] Shou Y, Ma Z, Lu T, Sorrentino BP. Unique risk factors for insertional mutagenesis in a mouse model of XSCID gene therapy. Proc Natl Acad Sci U S A. 2006;103(31):11730–5. Epub 20060724. doi: 10.1073/pnas.0603635103 ; PubMed Central PMCID: PMC1518804. 16864781 PMC1518804

