# Supplementary material for: Preclinical safety assessment of modified gamma globin lentiviral vector-mediated autologous hematopoietic stem cell gene therapy for hemoglobinopathies
Source: PLoS One. 2024 Jul 8;19(7):e0306719. doi: 10.1371/journal.pone.0306719 (PMC11230569; doi:10.1371/journal.pone.0306719)
Supplement: S1 Fig — (PDF) [file pone.0306719.s001.pdf]

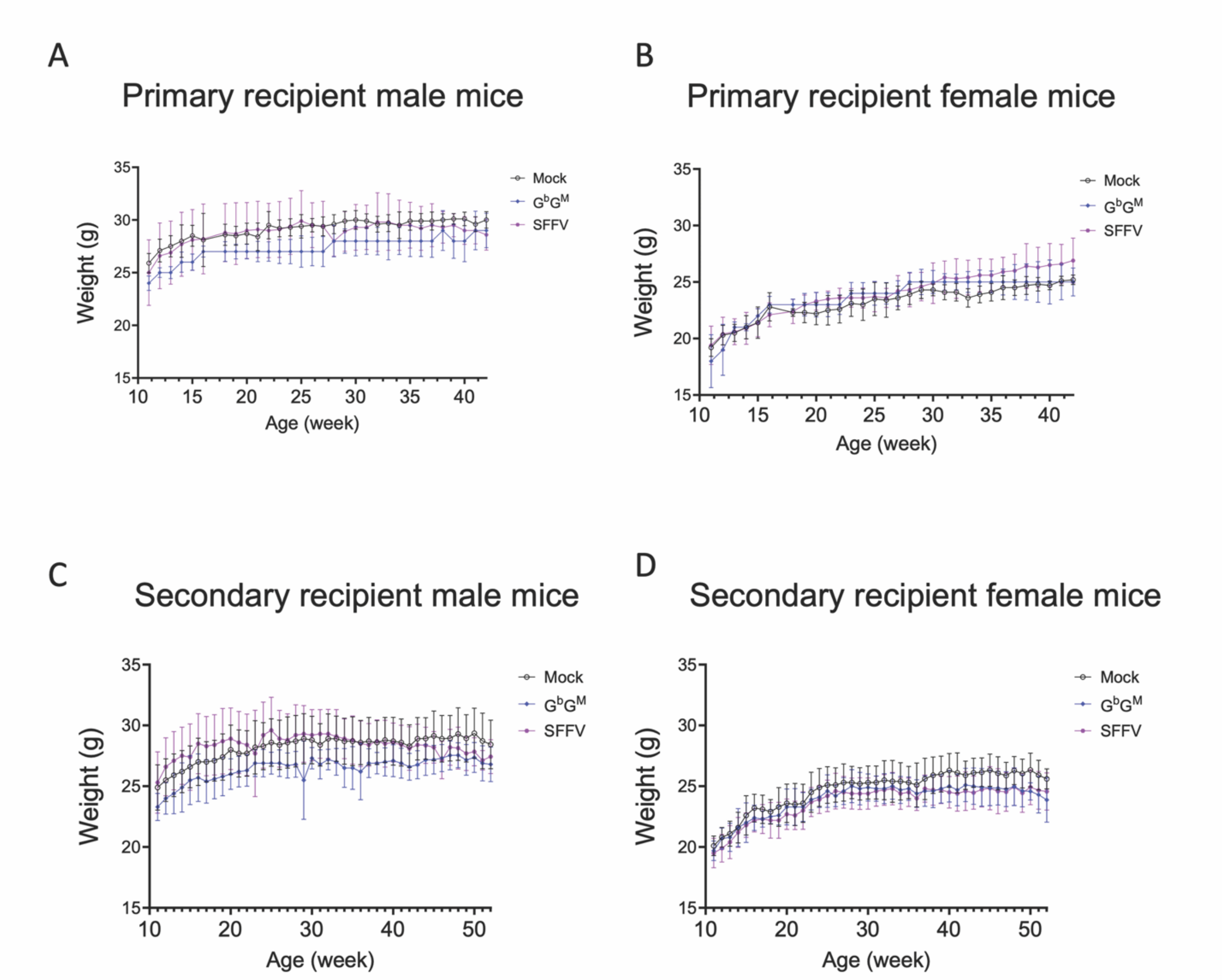


**S1 Fig. Longitudinal total body weight of male and female mice from the mock, $G^bG^M$, and SFFV treated group after primary and secondary BM transplants.** Body weight of primary male recipients (A) and female recipients (B) that were dosed with transduced HSPCs at week 10 of age and followed up until week 42. For the secondary transplant, male recipient mice (C) and female recipient mice (D) were dosed at week 9 and monitored until week 52. Each symbol represents the mean and standard deviation (SD) for each group at the specific time point.
