# Supplementary material for: Preclinical safety assessment of modified gamma globin lentiviral vector-mediated autologous hematopoietic stem cell gene therapy for hemoglobinopathies
Source: PLoS One. 2024 Jul 8;19(7):e0306719. doi: 10.1371/journal.pone.0306719 (PMC11230569; doi:10.1371/journal.pone.0306719)
Supplement: S2 Fig — (PDF) [file pone.0306719.s002.pdf]

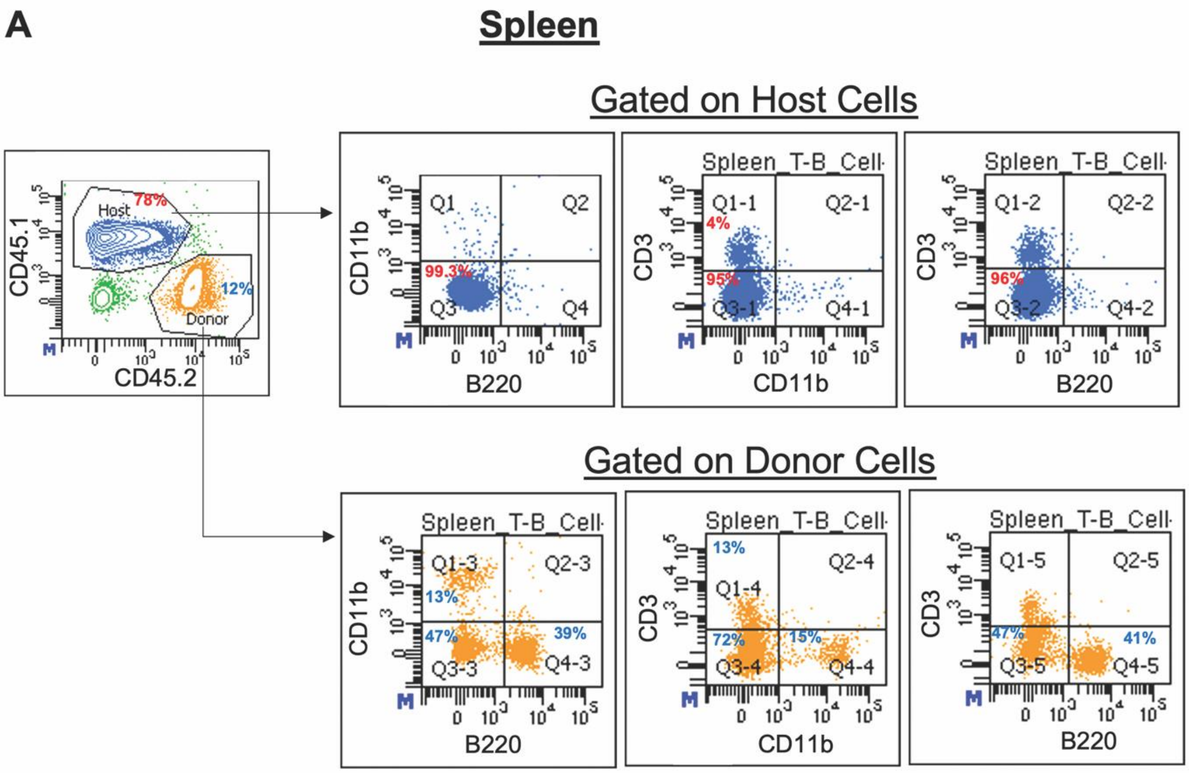

	CD45.1⁺ or CD45.2⁺	CD11b⁺ Myeloid	CD3⁺ T cells	B220⁺ B cells
Host CD45.1⁺ Cells	78%	~1%	4%	% <1%
Donor CD45.2⁺ Cells	12%	~13%	13%	~39%

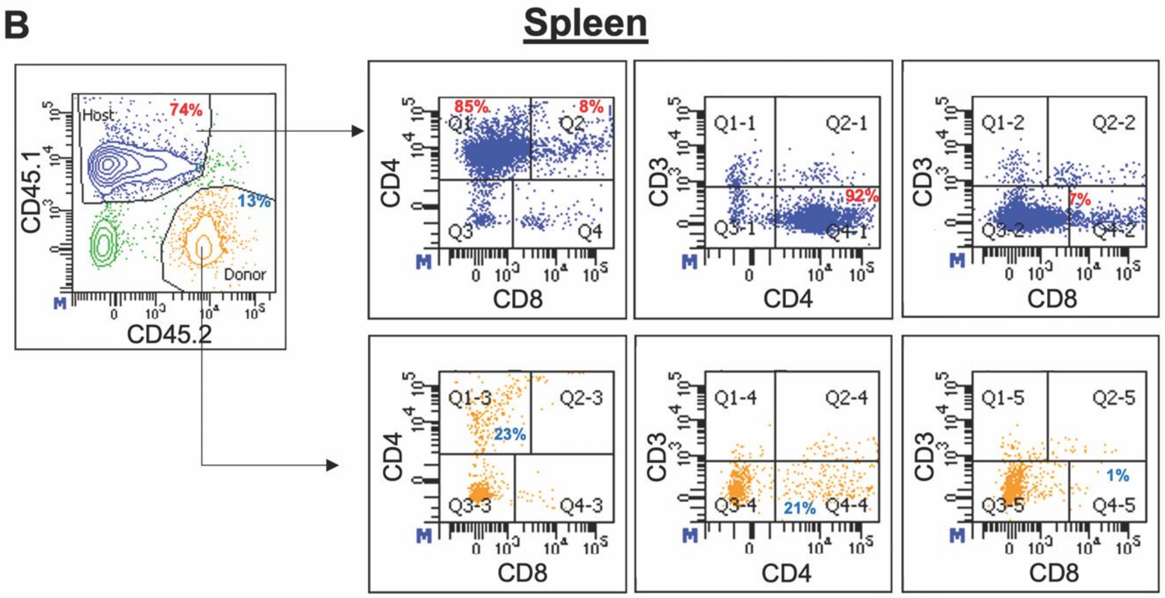

	$CD45.1^{+}$ or $CD45.2^{+}$	$CD3^{+}$ T cells	$CD4^{+}$ T cells	$CD8^{+}$ T cells
Host $CD45.1^{+}$ Cells	74%	~4.5%	93%	~10%
Donor $CD45.2^{+}$ Cells	13%	~5%	~24%	~3%

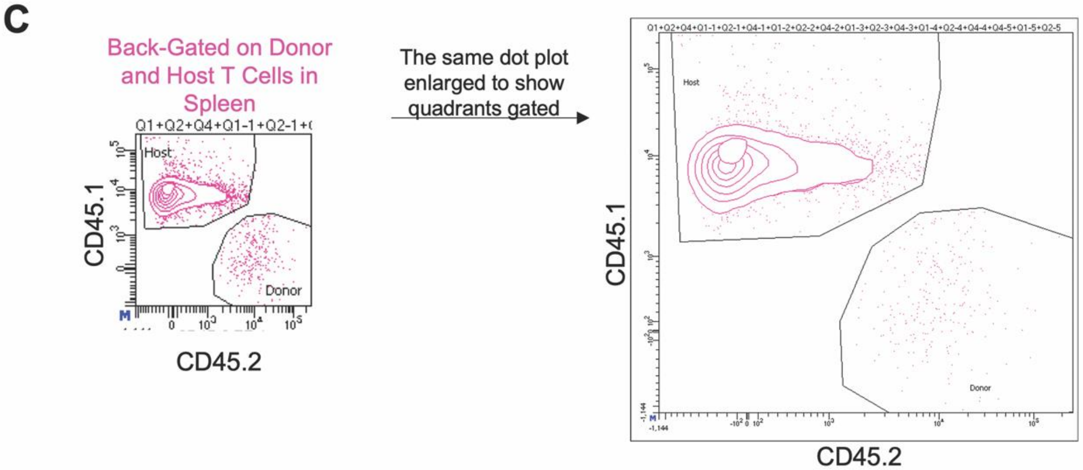

S2 Fig. **Engraftment and multi-lineage reconstitution in spleen of one lymphoma bearing secondary recipient mice.** Representative FACS plot of spleen cells from one of the secondary recipient animal with lymphoma (Mouse #6, please see Table 3). The mouse was injected with the bone marrow cells from primary recipient mice transplanted with $G^{b}G^{M}$ transduced HSPCs. (A-B) The FACS plot shows $CD45.1^{+}$ host and $CD45.2^{+}$ donor cell engraftment and the multi-lineage distribution of both host and donor cells in BOYJ secondary recipient mice. (A) Multi-lineages such as B lymphoid ($CDB220^{+}$), T lymphoid ($CD3^{+}$), myeloid ($CD11b^{+}$) were gated within

total CD45.1 $^+$ host and CD45.2$^+$ donor cells. (B) Sub-types of T cell lineages using CD3 $^+$, CD4 $^+$, CD8 $^+$ markers were gated within total CD45.1 $^+$ host and CD45.2$^+$ donor cells. A tabulated reconstitution (%) is presented below FACS plots. (C) The donor and host CD45.2 $^+$/CD45.1$^+$ engraftment plot is displayed to show the back-gated donor and host T cells in Spleen. The enlarged version of the same engraftment plot is shown on the right.
