# Supplementary material for: Preclinical safety assessment of modified gamma globin lentiviral vector-mediated autologous hematopoietic stem cell gene therapy for hemoglobinopathies
Source: PLoS One. 2024 Jul 8;19(7):e0306719. doi: 10.1371/journal.pone.0306719 (PMC11230569; doi:10.1371/journal.pone.0306719)
Supplement: S1 Table — (PDF) [file pone.0306719.s003.pdf]

S1 Table. Transplant and Engraftment After Primary Transplantation.

Time	Primary Recipients	Mock	G^bG^M	SFFV
**Predose**	# of mice transplanted	10	10	10
**Week 7**	# of live animals	10	9*	9*
	Mean PB VCN	0	1.5	2
	Mean CD45.2+ PB engraftment	92.7	91.3	91.8
**Week 17**	# of live animals	10	9	9
	Mean PB VCN	0	1.9	4.5
	Mean CD45.2+ PB engraftment	96.8	94.7	96.5

Abbreviations: Mock = mice transplanted with untransduced bone marrow cells; G^bG^M = recombinant γ-globin lentivirus vector with the G16D point mutation; SFFV = spleen focus-forming virus, PB=peripheral blood, VCN= vector copy number. * 1 animal died in each of these groups in <30 days from peri-transplant mortality
