# Supplementary material for: Preclinical safety assessment of modified gamma globin lentiviral vector-mediated autologous hematopoietic stem cell gene therapy for hemoglobinopathies
Source: PLoS One. 2024 Jul 8;19(7):e0306719. doi: 10.1371/journal.pone.0306719 (PMC11230569; doi:10.1371/journal.pone.0306719)
Supplement: S2 Table — (PDF) [file pone.0306719.s004.pdf]

S2 Table. Mean organ weights of the primary transplanted mice with Mock, GbGM, and SFFV at 8 months post-transplant.

Weights (g)								
Group/ Tissue		Thymus	Lungs	Liver + Gallbladder	Spleen	Kidney	Lymph Nodes	Heart
Mock	Mean	0.05	0.24	1.53	0.09	0.44	0.09	0.16
	Std. Dev.	0.01	0.05	0.30	0.02	0.06	0.05	0.02
	n	10	10	10	10	10	10	10
G^bG^M	Mean	0.04	0.26	1.54	0.09	0.48	0.10	0.16
	Std. Dev.	0.01	0.04	0.28	0.02	0.08	0.04	0.03
	n	8	8	8	8	8	8	8
SFFV	Mean	0.04	0.24	1.54	0.08	0.41	0.09	0.15
	Std. Dev.	0.01	0.05	0.19	0.01	0.05	0.04	0.02
	n	9	9	9	9	9	9	9
