# Supplementary material for: Preclinical safety assessment of modified gamma globin lentiviral vector-mediated autologous hematopoietic stem cell gene therapy for hemoglobinopathies
Source: PLoS One. 2024 Jul 8;19(7):e0306719. doi: 10.1371/journal.pone.0306719 (PMC11230569; doi:10.1371/journal.pone.0306719)
Supplement: S3 Table — (PDF) [file pone.0306719.s005.pdf]

**S3 Table. Mean organ weights of the secondary transplanted mice with Mock, GbGM, and SFFV at 10 months post-transplant.**

| Weights (g) | | | | | | | | |
|---|---|---|---|---|---|---|---|---|
| Group/ Tissue | | Thymus | Lungs | Liver + Gallbladder | Spleen | Kidney | Lymph Nodes | Heart |
| Mock | Mean | 0.04 | 0.26 | 1.61 | 0.11 | 0.49 | 0.17 | 0.17 |
| | Std. Dev. | 0.02 | 0.06 | 0.30 | 0.05 | 0.08 | 0.11 | 0.02 |
| | n | 20 | 20 | 20 | 20 | 20 | 20 | 20 |
| $G^bG^M$ | Mean | 0.06 | 0.24 | 1.62 | 0.14 | 0.52 | 0.11 | 0.17 |
| | Std. Dev. | 0.05 | 0.03 | 0.48 | 0.11 | 0.17 | 0.08 | 0.03 |
| | n | 16 | 16 | 16 | 16 | 16 | 16 | 16 |
| SFFV | Mean | 0.04 | 0.25 | 1.81 | 0.21* | 0.52 | 0.11 | 0.19 |
| | Std. Dev. | 0.02 | 0.04 | 0.36 | 0.17 | 0.10 | 0.09 | 0.04 |
| | n | 13 | 13 | 13 | 13 | 13 | 13 | 13 |
