# Supplementary material for: Preclinical safety assessment of modified gamma globin lentiviral vector-mediated autologous hematopoietic stem cell gene therapy for hemoglobinopathies
Source: PLoS One. 2024 Jul 8;19(7):e0306719. doi: 10.1371/journal.pone.0306719 (PMC11230569; doi:10.1371/journal.pone.0306719)
Supplement: S4 Table — A. Hematological analyses of WT BoyJ recipient mice following the transplant with GbGM, Mock, and SFFV transduced HSPC 8 months following primary transplant. B. Hematology analyses of secondary WT BoyJ recipient mice following the injection of BM of primary mice transplanted with GbGM, mock, and SFFV transduced HSPCs 10 months post-secondary transplant. (PDF) [file pone.0306719.s006.pdf]

S4A Table. Hematological analyses of WT BoyJ recipient mice following the transplant with G^bG^M, Mock, and SFFV transduced HSPC 8 months following primary transplant.

Primary Recipients		G^bG^m			Mock			SFFV		
Param.	Units	N	Mean	SD	N	Mean	SD	N	Mean	SD
WBC	K/µL	9	7.58	1.58	10	12.59	1.76	7	10.25	2.58
NE#	K/µL	9	1.83	0.69	10	2.52	0.61	7	1.79	0.74
LY#	K/µL	9	5.42	1.18	10	9.78	1.33	7	8.24	2.11
MO#	K/µL	9	0.26	0.11	10	0.24	0.13	7	0.21	0.09
EO#	K/µL	9	0.02	0.01	10	0.04	0.04	7	0.01	0.00
BA#	K/µL	9	0.00	0.00	10	0.01	0.02	7	0.00	0.00
NE%	%	9	24.1	5.52	10	19.84	3.52	7	17.28	5.15
LY%	%	9	72.04	5.67	10	77.85	3.83	7	80.42	4.37
MO%	%	9	3.55	1.72	10	1.91	0.87	7	2.20	1.14
EO%	%	9	0.27	0.14	10	0.33	0.39	7	0.08	0.03
BO%	%	9	0.03	0.03	10	0.07	0.16	7	0.02	0.02
RBC	M/µL	9	9.08	0.51	10	9.16	0.55	7	9.66	0.30
HB	g/dL	9	12.2	1.0	10	12.5	0.9	7	12.8	1.5
HCT	%	9	46.4	2.8	10	46.2	2.7	7	49.3	2.4
MCV	fL	9	51.1	2.4	10	50.5	2.0	7	51.0	1.7
MCH	pg	9	13.5	0.7	10	13.7	0.8	7	13.2	1.2
MCHC	g/dL	9	26.4	1.6	10	27.1	1.8	7	26.0	2.2
RDW	%	9	18.7	1.5	10	17.7	1.0	7	18.0	0.5
PLT	K/µL	9	785	148	10	923	165	7	915	70
MPV	fL	9	4.9	0.2	10	4.8	0.3	7	4.9	0.2

Red Blood Cell Count (RBC), White Blood Cell Count (WBC), Hemoglobin (HGB), Hematocrit (HCT), Mean Corpuscular Volume (MCV), Mean Corpuscular Hemoglobin Concentration (MCHC), Platelet Count (PLT). Mean Corpuscular Hemoglobin (MCH), Mean Platelet Volume (MPV), Red Cell Distribution Width (RDW),

Absolute Cell Counts: Neutrophils NE#, Lymphocytes LYM#, Monocytes MO#, Eosinophils EO#, Basophils BA#. WBC Differential counts are marked by %. For the units: $K=10^3$ and $M=10^6$

S4B Table. Hematology analyses of secondary WT BoyJ recipient mice following the injection of BM of primary mice transplanted with GbGM, mock, and SFFV transduced HSPCs 10 months post-secondary transplant.

**Primary Recipients**		**G^bG^m**			**Mock**			**SFFV**		
Param.	Units	N	Mean	SD	N	Mean	SD	N	Mean	SD
WBC	K/μL	16	8.24	9.73	20	10.31	3.48	13	29.58	46.67
NE#	K/μL	16	3.30	5.47	20	2.66	0.85	13	15.86	30.31
LY#	K/μL	16	3.95	2.70	20	7.33	3.17	13	9.07	8.69
MO#	K/μL	16	0.63	1.63	20	0.30	0.15	13	3.51	9.37
EO#	K/μL	16	0.30	1.15	20	0.02	0.02	13	1.11	2.51
BA#	K/μL	16	0.06	0.23	20	0.00	0.01	13	0.05	0.10
NE%	%	16	35.57	12.22	20	27.96	11.51	13	37.67	18.71
LY%	%	16	55.76	16.73	20	68.00	13.52	13	54.86	23.78
MO%	%	16	5.46	4.08	20	3.16	1.80	13	6.14	6.35
EO%	%	16	1.04	2.71	20	0.23	0.36	13	1.25	1.89
BO%	%	16	0.18	0.53	20	0.06	0.14	13	0.08	0.15
RBC	M/μL	16	9.25	1.34	20	9.67	1.21	13	7.33	2.34
HB	g/dL	16	12.3	1.4	20	12.5	1.3	13	10.4	2.2
HCT	%	16	41.3	7.5	20	40.3	4.7	13	35.1	7.5
MCV	fL	16	45.2	9.3	20	41.7	2.3	13	50.4	9.9
MCH	pg	16	13.4	1.1	20	12.9	0.4	13	14.9	2.6
MCHC	g/dL	16	30.2	3.0	20	31.0	1.5	13	29.8	2.6
RDW	%	16	20.0	4.7	20	17.7	1.1	13	20.3	3.7
PLT	K/μL	16	819	329	20	1069	230	13	887	450
MPV	fL	16	5.4	0.6	20	4.9	0.4	13	5.4	0.6

Red Blood Cell Count (RBC), White Blood Cell Count (WBC), Hemoglobin (HGB), Hematocrit (HCT), Mean Corpuscular Volume (MCV), Mean Corpuscular Hemoglobin Concentration (MCHC), Platelet Count (PLT). Mean Corpuscular Hemoglobin (MCH), Mean Platelet Volume (MPV), Red Cell Distribution Width (RDW),

Absolute Cell Counts: Neutrophils NE#, Lymphocytes LYM#, Monocytes MO#, Eosinophils EO#, Basophils BA#. WBC Differential counts are marked by %. For the units: $K=10^3$ and $M=10^6$
